# Serological Responses to Influenza Vaccination during Pregnancy

**DOI:** 10.3390/microorganisms9112305

**Published:** 2021-11-06

**Authors:** Ana Vazquez-Pagan, Stacey Schultz-Cherry

**Affiliations:** 1 Graduate School of Biomedical Sciences, St. Jude Children’s Research Hospital, Memphis, TN 38105, USA; ana.vazquez-pagan@stjude.org; 2 Department of Infectious Diseases, St. Jude Children’s Research Hospital, Memphis, TN 38105, USA

**Keywords:** influenza vaccine, pregnancy, placenta, passive immunity, maternal antibody interference, early life immunity, immunological blunting

## Abstract

Pregnant women, newborns, and infants under six months old are at the highest risk of developing severe and even fatal influenza. This risk is compounded by the inability to vaccinate infants under six months, highlighting the importance of vertically transferred immunity. This review identifies novel insights that have emerged from recent studies using animal models of pregnancy and vaccination. We also discuss the knowledge obtained using existing clinical trials that have evaluated influenza-specific serological responses in pregnant women and how these responses may impact early life immunity. We delineate the mechanisms involved in transferring specific maternal antibodies and discuss the consequences for early life immunity. Most importantly, we highlight the need for continued research using pregnant animal models and the inclusion of pregnant women, a commonly neglected population, when evaluating novel vaccine platforms to better serve and treat communicable diseases.

## 1. Introduction

Influenza viruses are enveloped, negative sense, single-stranded RNA viruses that belong to the family Orthomyxoviridae. Influenza A Viruses (IAV) are responsible for most seasonal cases and outbreaks of influenza. Distinct hemagglutinin (HA) and neuraminidase (NA) define the numerous IAV subtypes. Influenza B viruses (IBV) are also associated with two seasonal epidemics [1,2,3]. Unlike IAV, IBVs are not divided into subtypes. Instead, the following two genetically distinct lineages circulate in humans: B/Victoria/2/87-like (Victoria lineage) and B/Yamagata/16-88-like (Yamagata lineage) [1,4]. While most individuals infected with the influenza virus experience mild symptoms and do not require hospitalization, there are vulnerable subsections of the population who are at a higher risk of developing severe infections, including pregnant women [5,6,7].

Pregnancy has been acknowledged as a risk factor for severe complications from various infectious agents for nearly a century [8]. For example, epidemiological and empirical studies suggest that influenza infection can result in up to a five-fold increase in cardiopulmonary complications compared to non-pregnant women [6]. Mortality rates were also highest among pregnant women (as high as 45%) during major influenza pandemics (1918, 1957, 1968, and 2009) [5,6,9,10]. In addition, the infection-related complications extend to the offspring, with a well-known increased risk of miscarriage, stillbirth, neonatal death, preterm birth, and low birth weight neonates [5,10,11,12,13,14,15,16,17,18]. Given the myriad of adverse outcomes that can result from influenza infection, prevention through vaccination is the best control strategy [19].

The vaccination of pregnant women protects both the mother and the newborn [20,21], and the immunization of pregnant women is highly recommended and safe [22]. Generally, immunization would be the best strategy to protect this subsection of the population. The transplacental transfer of maternal antibodies during pregnancy and breastfeeding is crucial for the infant’s protection against infections [22,23,24]. Previous studies have shown that the vaccination of pregnant women against influenza protects their young infants from laboratory-confirmed influenza infection [21,25,26].

Despite its clear benefits, only 50% of women in the U.S. were vaccinated either before (15.3%) or during pregnancy (35.0%) in 2015 [27]. Since infants cannot be vaccinated against influenza before six months of age, it is advantageous to confer a robust humoral response via the transplacental transfer of maternal antibodies while in utero or by breast milk [20,21]. This review highlights the need for continued research using pregnant animal models and for the consideration of pregnant women when evaluating novel vaccine platforms to better serve and treat communicable diseases.

### Vertically Transferred Immunity during Pregnancy: Main Mechanisms and Mediators

**Figure 1:**
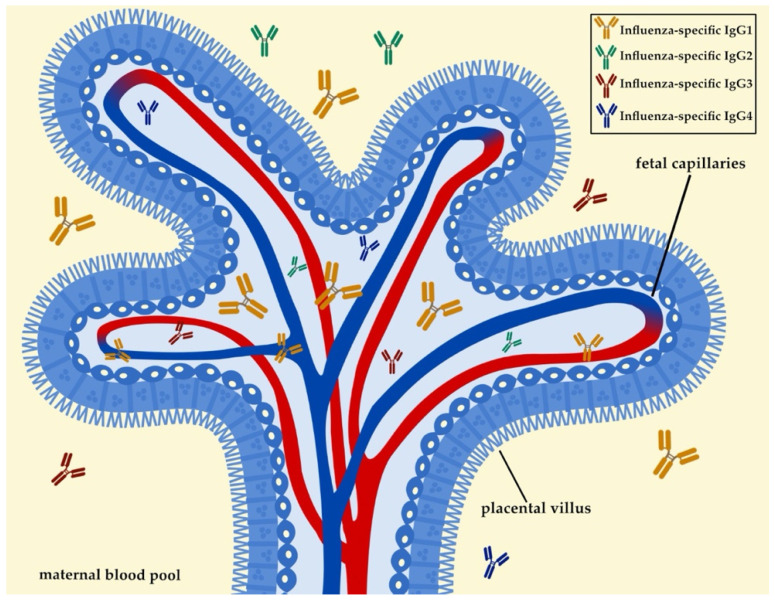
One of the main mechanisms of vertically transferred immunity during pregnancy is the transplacental transfer of IgG. IgG1 is the main subtype that is predominantly transferred to the infant, with other subtypes being transferred to a lesser degree. The transport of IgG across the placental barrier is mediated by the FcRn. FcRn is primarily expressed in placental syncytiotrophoblasts, the outermost layer of the placental villus. This figure was created with Bio Render, Inkscape, and Affinity Designer.

The dynamics of vertically transmitted immunity are crucial, as they provide a layer of protection to the neonate from infections during early life [20,28,29,30,31,32,33]. Besides posing as a barrier against infections, the placenta also mediates the transplacental transfer of maternal antibodies throughout pregnancy [34]. The neonatal Fc-receptor (FcRn) plays a vital role in mediating the transplacental transfer of maternal-pathogen-specific antibodies. FcRn is primarily expressed in placental syncytiotrophoblasts, and it binds to the Fc fragment of IgG antibodies [35,36]. This binding activity assists with the transportation of IgG antibodies to the specific sites where immunity is needed. Interestingly, the amount of antibody that can be vertically transferred depends on the amount of FcRn expressed by the placental syncytiotrophoblasts [28,29,37,38] (Figure 1). While IgG is the primary antibody transported during pregnancy, not all subtypes and IgG subclasses are equally transferred. For example, FcRn is biased towards IgG1, and the efficacy of other subtypes such as IgG4, IgG3, and IgG2 is generally reduced (Figure 1). The bias towards IgG1 is beneficial as most IgG antibodies targeting influenza virus are of the IgG1 subtype [37,38,39,40,41].

Transplacental antibody transport occurs early in the pregnancy and reaches its peak during the last month of the pregnancy (or the last four weeks of gestation) [24,31]. Albrecht et al. speculated that an increased growth of the placenta could account for the increase in FcRn expression and, subsequently, a higher antibody transport rate [Reviewed in [23]]. Besides FcRn expression, another predictive factor of transplacental antibody transfer is the level of maternal antibody present [24]. The progression of the pregnancy, recent immunizations, a well-balanced maternal nutritional status, and a male gender of the newborn have all been shown to increase maternal antibody presence in the infant [23,32,42,43]. Interestingly, one study found that vitamin A could influence maternal antibody transfer following maternal influenza vaccination [44]. However, data are based on a small sample size, and it could be argued that these results are speculative.

**Figure 2:**
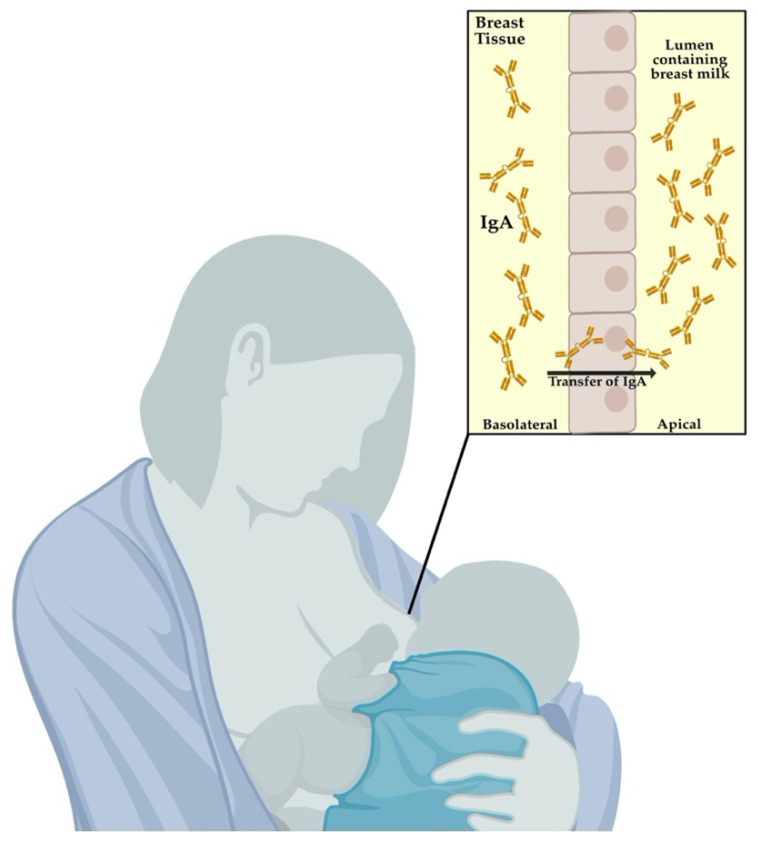
Another crucial mechanism of vertically transferred immunity during pregnancy is the transfer of secretory IgA via breast-milk post-birth. IgA1 and IgA2 are the main subtypes transferred to the infant, with IgA1 being crucial for protection against influenza virus infection. This figure was created with Bio Render and Affinity Designer.

Besides the transplacental transfer of maternal antibodies, the intake of breast milk during early life can also provide infants with a substantial amount of secretory IgA (Figure 2). IgA antibodies are produced by plasma cells found in tissues and sites associated with mucosal surfaces such as the mammary gland or the respiratory epithelia. IgA1 and IgA2 are the main subclasses of IgA present in humans [45]. IgA1 is present in the respiratory tract, saliva, serum, and skin. IgA2 is the primary secretory antibody of the intestine. As previously discussed by Hanson and Weinberg [46], it is essential to note that breast milk IgA is not absorbed by the infant’s gut. Instead, breast milk IgA can coat the mucosal surface of the intestine to protect it from pathogens. Such findings have led multiple studies to highlight the acquisition of IgA antibodies through breastfeeding as a benefit for newborn and infant health [39,40,41,47]; (reviewed in [23]). For example, there is an enhanced transfer of influenza-specific and neutralizing IgA upon maternal influenza vaccination to the neonate. These findings were also associated with a reduction in respiratory illnesses of the infants during the first six months of life [47]. Future studies should consider investigating the levels of breast-milk IgA in newborns and infants.

## 2. Maternal Antibody Interference: The Downside of Passively Acquired Immunity

While maternal vaccination may be the best strategy for protecting the offspring during early life, several studies have shown that maternal antibodies may have a negative impact on active immunization [48,49,50,51] as summarized in [23]. For example, infants that acquire high levels of maternal antibodies will not mount the immune response required, due to routine immunizations. This inhibitory effect is also known as immunological blunting and has been shown to occur with multiple maternal immunizations, including measles, influenza, and pertussis [37]. 

Immunological blunting involves cross-reactivity between the B-cell receptor (BCR) and the Fcγ receptor FcγRIIB [37,52], both of which are expressed on the surface of the B-cells. Pathogen-specific maternal antibodies can recognize pathogen-specific epitopes with a unique affinity to each BCR. Once infants are immunized, pathogen fragments enter the circulation. BCR and maternal antibodies can bind these pathogen-specific fragments at the same time. This event can lead to the creation of contradicting signals within the B-cell. At this point, the BCR recognizes the new antigen, and it produces a signal that leads to plasma cell differentiation and antibody production. FcγRIIB exalts the presence of a specific maternal antibody to the antigen and subsequently inhibits any further antibody production. This can result in the inhibition of a stimulatory BCR signal and can further hamper antibody production in the infant’s immune system [37,52]; reviewed in [23]. Much of our understanding of the impact of the influenza vaccination on mother and offspring derives from animal studies. 

## 3. Animal Studies: Pregnancy and Vaccination

The influenza virus replicates in the respiratory epithelium, eliciting both pro-inflammatory and anti-viral responses, which are required to control infection [7,53]. Since influenza disease can be severe during pregnancy [5,6,9,53,54,55], vaccination during pregnancy becomes crucial to protect the mother and the infant during the first months of life. Given the difficulties in performing extensive vaccine studies with longitudinal sampling, animal models of pregnancy have provided an opportunity to establish a scientific premise to move influenza vaccine candidates to the clinic. This section summarizes animal models of pregnancy that elucidate the outcomes of maternal influenza vaccination in protecting the mother and influencing the offspring’s humoral immunity landscape. We also propose potential ways forward and questions that could be used to follow up on the results from these studies. 

### 3.1. Mice 

Mice and humans both possess a hemochorial placenta, which acts as the main barrier that mediates antibody transfer. While there are several differences in placentation between mice and humans that should be considered, there are also similarities that allow us to evaluate the mechanisms of passive immunity in a pregnant mouse model [Reviewed in [56]]. Studies that use the mouse model have been instrumental in further defining the mechanisms involved in vertically transferred immunity [Reviewed in [23]]. 

Studies have evaluated the vaccine’s efficacy of inducing HA-specific antibodies using the standard of care vaccination platforms such as the seasonal trivalent vaccine (TIV) in a BALB/c pregnant mouse model [57,58]. Van der Lubbe et al. varied the regimen of maternal immunization and evaluated how this shifted the levels of maternal antibodies on the progeny [56]. Overall, the authors found that a prime-boost method provided higher antibody titers by HA-specific ELISA in the dams compared to a single immunization. This trend was also consistent in the offspring, where multiple maternal immunizations increased their antibody titers compared to a single shot. The longevity of maternal antibodies also correlated with the levels of antibodies transferred. While a single vaccination would lead to undetectable antibody levels in the pups five weeks after birth, a prime-boost vaccination regimen provided readily detectable antibodies at seven weeks post-birth [56]. 

Overall, these data suggest that a prime-boost method with a TIV platform offers a higher proportion of antibody titers in the dams and their offspring as compared to a single dose. While the studies evaluated the protection from influenza in the offspring born to vaccinated dams, the authors did not evaluate protection from challenge in the vaccinated dams. Future studies should evaluate the functionality of the antibodies to protect from influenza. These studies should also assess whether the increased levels of HA-specific antibodies correlate with survival after a lethal challenge in the dams and not solely the offspring. BALB/c mice are the predominant mouse strain used for vaccination studies during pregnancy. Thus, it is essential to consider how data using additional mouse strains such as C57BL/6J.

Other studies have explored different vaccine platforms with a high translational capacity in developing countries with a greater limit of resources [59]. Such platforms include skin immunization using microneedle (MN) patches. These have shown great promise in providing a more potent and longer-lasting immune response compared to more conventional vaccination platforms [60,61]. In Esser et al., the authors fabricated MN patches to deliver a subunit seasonal influenza vaccine in the skin of pregnant mice compared to non-pregnant controls. Immunization via an MN patch induced higher levels of influenza antibody titers, including IgG, IgG1, and IgG2a specific antibodies in pregnant mice, albeit the levels were lower than in non-pregnant mice. These findings can translate to protection from a lethal challenge. In addition, authors observed higher survival rates in pregnant and non-pregnant mice that received MN patch vaccination than for the intramuscular (IM) and intradermal (ID) immunized groups. Interestingly, there was a strong correlation between hemagglutination inhibition (HAI) titers and protection in the pregnant dams vaccinated with MN patches. This trend is not consistent for the non-pregnant dams, which suggests an involvement of additional humoral and cellular responses in this group [57,58,59,60]. 

The authors also evaluated passive immunity in the offspring of MN patch-vaccinated mice. While the influenza-specific antibodies in the offspring born to dams that received IM or ID immunization decreased over time, offspring born to MN patch-vaccinated dams had higher whole-virus specific IgG ELISA titers until eight weeks of age. To test whether passively transferred maternal influenza antibodies were protective, 6-week-old offspring born to IM-, ID- and MN-patch-vaccinated dams were challenged with a lethal dose of a mouse-adapted homologous influenza virus. Offspring born to MN patch-vaccinated dams had the highest survival rate (~40%), which correlated with the levels of neutralizing antibodies. These results were in stark contrast to pups born to ID- and IM-immunized dams, who all succumbed to the infection. Since offspring were not vaccinated, these results suggest that MN patch-induced maternal antibodies are longer-lived and persist for at least 6 weeks after birth [58,59]. These studies provide valuable insights and potential for platforms that can protect infants until they are able to get vaccinated. Future studies are needed to evaluate the breadth and depth of these antibodies and B-cell mediated immunity. Understanding whether these innovative platforms can also provide cross-reactive immunity to distinct seasonal influenza strains is an intriguing and essential line of inquiry. 

While many studies utilizing a pregnant mouse model have evaluated the protection against a lethal challenge from H1N1 and H3N2 influenza strains, fewer studies have evaluated vaccine candidates for highly pathogenic influenza strains such as H5N1. In Hwang et al., pregnant dams were immunized with an inactivated H5N1 influenza vaccine [61]. To evaluate protection from lethal challenge, dams received one or two doses of the inactivated H5N1 influenza vaccine and underwent a lethal homologous challenge with unimmunized mice serving as negative controls. Pregnant dams immunized with two doses of the inactivated H5N1 influenza vaccine had a 100% survival rate. However, pregnant mice vaccinated with one dose or unimmunized dams had between 60% and 0% survival rates, respectively. Viral titers were not detected in tissues of pregnant mice immunized with two doses. Conversely, viral titers were detected in the lung, heart, rectum, and brain tissues of pregnant mice vaccinated with one dose and unimmunized pregnant dams [61]. 

The authors also investigated whether the vaccine was also protective against a lethal heterologous challenge. Unimmunized pregnant mice and those immunized with one dose dams were not protected from a heterologous lethal challenge with H5N1 influenza viruses. While pregnant dams that were vaccinated with two doses had a 100% survival rate upon homologous challenge, they were only protected by up to 30% from the heterologous challenge. These findings suggest that maternal vaccination improves survival and limits pathogenicity upon a lethal homologous challenge. More specifically, two doses of an inactivated H5N1 influenza vaccine appear to elicit a more protective response and two doses of inactivated vaccine of H5N1 influenza viruses were needed to provide minimal cross-reactive immunity against a lethal heterologous challenge [61]. 

Maternal H5N1 influenza infection has also been shown to be detrimental to the developing fetus. Highly pathogenic strains of the influenza virus, such as H5N1, can trespass the placental barrier due to the cleavability of the H5 HA by furin resulting in increased cellular tropism, systemic spread, and enhanced pathogenicity [17,62]. The authors evaluated the levels of detectable virus in the fetal compartment. Upon a homologous lethal challenge, fetuses from dams immunized with two doses had no detectable virus while 6/15 and 15/15 fetuses had a detectable virus from dams vaccinated with one dose or unimmunized dams, respectively. Upon a heterologous challenge, all fetuses from dams immunized with a single dose or unimmunized dams had high levels of detectable virus, while 10/15 fetuses born from pregnant dams that were vaccinated with two doses had viral titers. These data suggest that maternal vaccination may be critical for not only protecting the pregnant mother from highly pathogenic influenza strains but also the offspring [61]. However, these data also imply that this protection is limited to strains that are homologous to that of the vaccine [61]. Future studies should evaluate the ability of other vaccination platforms to protect the mother and offspring against highly pathogenic strains.

### 3.2. Ferrets 

Ferrets remain the gold standard to study influenza pathogenesis [63,64,65]. Ferrets are naturally susceptible to infection with a wide variety of viruses and display influenza-like illness (ILI) symptoms similar to those observed in humans due to an analogous distribution of sialic acid residues within the respiratory tract [18,64]. Although there are limitations associated with the ferret model, including a dearth of reagents, the National Institute of Allergy and Infectious Diseases (NIAID)-funded Centers for Excellence in Influenza Research and Surveillance has launched an initiative to develop ferret immune reagents and antibodies to obtain further knowledge using the ferret model [65]. As promising new vaccine platforms emerge, there is a need to test vaccine candidates in a model that best recapitulates influenza’s transmission dynamics and disease severity. Herein, we summarize and comment on contemporary studies that have been conducted using a pregnant ferret model to evaluate the disease severity of the pregnant ferret and passive immunity to influenza vaccination and infection in young kits [64,66,67,68,69,70]. 

Due to the observed pathogenicity of the 2009 influenza A H1N1 pandemic (H1N1pdm) in adult ferrets, Amorsolo et al. evaluated the kinetics of influenza-specific antibodies from natural infection and passive immunity in a pregnant ferret model. Kits, that were born to ferrets naturally infected with H1N1pdm, had detectable HAI titers. However, antibody levels decreased substantially after 6-weeks of age. To understand the breadth of these antibodies, naïve kits, and kits with maternal H1N1pdm antibodies were infected with A/South Dakota/6/2007 (SD07, H1N1), A/Gilroy/231/2011 (Gil11, H1N1pdm), and A/Rhode Island/1/2010 (RI10, H3N2) influenza viruses. Upon homologous challenge, kits with maternal antibodies had a significantly reduced viral load in the lungs. Conversely, a heterologous challenge in kits with maternal antibodies did not result in any differences in the viral burden compared to naïve kits of the negative control group, suggesting that protection conferred by influenza-specific maternal antibodies was limited and highly strain-specific [71]. 

Similar to the mouse model, studies have also shown that long-lived maternal antibodies may have a negative impact on active immunization in the ferret model [48,49,50,51]. Given that maternal antibodies conferred by natural infection were not as protective upon heterologous challenge, the authors evaluated whether these maternal antibodies interfered with influenza vaccine immunogenicity. To understand whether this was the case, naïve kits and kits with maternal antibodies were immunized with a live-attenuated virus vaccine (LAIV) via a prime-boost regimen. Naïve ferrets had higher levels of H1N1pdm specific antibodies compared to kits that had a passive transfer of maternal antibodies via natural infection. Interestingly, this result was only found upon homologous challenge. When infected with a different strain or sub-type, the serological responses were higher, even in kits that had a passive transfer of H1N1pdm specific maternal antibodies [71]. 

To further evaluate the functionality of maternal antibodies, a normal ferret serum, a hyperimmune ferret serum used against H1N1pdm (HAI titer of 2048), and a 1:8 dilution of hyperimmune ferret serum were passively administered via intravenous injection to naïve kits. The authors observed that the baseline HAI responses in naïve ferrets for whom the serum was passively transferred, mimicked those of kits with maternal antibodies. After their passive transfer with hyperimmune ferret serum and immunization with LAIV, kits that had received a homologous vaccine strain had the lowest HAI titers as compared to kits that were immunized with a heterologous vaccine strain (H3N2 or Influenza B). These results suggest that maternal antibodies can cross-react with the immunizing influenza strain when using a LAIV platform. This interference could dampen the humoral immune response against the strain of interest. A consideration of the time at which the maternal antibodies entirely waned may prove to be crucial in maximizing cross-reactive humoral immunity during early life [72]. 

The ferret model is often used to evaluate the transmission dynamics of the influenza virus due to similarities between the ferret and human respiratory systems [64,65]. Paquette et al. further investigated influenza virus dynamics in the mother-infant dyad using influenza-infected neonatal ferrets [52]. Inoculation of young kits with A/California/07/2009 H1N1(A/CA/07/09 H1N1) during the lactation stage resulted in influenza transmission to the maternal lung and mammary gland tissue. Consequently, the infection led to the cessation of milk production and, eventually, maternal death [71]. All infected kits also succumbed to the infection. Furthermore, the inoculation of lactating ferrets resulted in all the infant ferrets succumbing to the infection [71] whereas, all lactating mothers survived [71]. These results suggest that some influenza strains may be capable of replicating in the mammary gland tissue. Future studies should evaluate whether additional influenza strains have similar dynamics of transmission or whether this phenomenon is unique to the A/CA/07/09 H1N1 strain used in these studies. Additionally, these studies highlight the lactating ferret as a potential model to study the mother-child dyad transmission dynamics, including maternal antibody transfer.

## 4. Insights from Vaccination Studies against Influenza during Pregnancy 

Pregnancy is composed of multiple stages, each characterized by its own inflammatory milieu [72]. Adaptations of the maternal immune system occur during pregnancy to accommodate the development of a foreign fetus. Epidemiological and empirical studies have established that influenza illness during pregnancy is associated with increased disease severity [5,6,9,10,15,19,54,73]. To this end, the World Health Organization deemed pregnant women as one of the priority groups for the influenza vaccine [19], as vaccination also protects newborns during the first years of life [21,25,26,74]. These findings continue to demonstrate the importance of receiving the influenza vaccination during pregnancy. During the 2016–2017 influenza season, 53.6% of women responding to an internet panel survey conducted between 28 March and 7 April 2017 reported that they received the influenza vaccine before or during pregnancy [75]. Although these numbers reflect significant progress, further improvement is necessary to meet the U.S. Health and Human Services’ Healthy People 2020 goal of vaccinating 80% of pregnant women against influenza [76]. 

Currently, the Advisory Committee on Immunization Practices (ACIP) and the American College of Obstetricians and Gynecologists recommend that all women who are pregnant or who might be pregnant or postpartum during the influenza season receive the influenza vaccine [54]. Influenza vaccination can be administered at any time during pregnancy, before and during the influenza season. Any licensed, recommended, and age-appropriate TIV may be used. LAIV is not recommended for use during pregnancy. In the sections below, we summarize clinical trials investigating long-lived antibody protection post-vaccination, the functionality of antibody responses elicited via immunization, and vertically transferred immunity in infants born to TIV-immunized mothers.

### 4.1. Maternal Influenza Vaccination 

It has been previously reported that TIV is safe and 50% efficacious in Human Immunodeficiency Virus (HIV)-uninfected pregnant women and 49% efficacious in their infants until 24-weeks postpartum (or six months old) [77]. Further knowledge on the evaluation of the duration and persistence of antibody levels following immunization in a cohort of pregnant women is still required. However, one study showed that 70% of vaccinated women maintained HAI antibody titers (HAI > 1:40) to at least one vaccine strain for up to eight months post-vaccination [78]. Other studies also assessed the persistence of HAI antibodies to 3 influenza strains included in the TIV administered to pregnant women during the 2011 influenza season [79]. In the latter study, the study also evaluated the efficacy of TIV-mediated responses against PCR-confirmed influenza infection in the following 2012 influenza season [79].

The study concluded that in a vaccine naïve population, persistent protective effects were observed during two successive influenza seasons with similar circulating strains, which were matched with the strains of the vaccine formulation [79]. TIV was shown to provide a sustained, HAI-mediated response, including the persistence of seroprotective antibodies for up to a year [80]. The trend included long-term protection against antigenically similar influenza strains for up to nineteen months [79]. One year after vaccination, the majority of women who received TIV during pregnancy had HAI titers above the putative threshold for protection against influenza illnesses. Others also evaluated the efficacy of the influenza vaccine in cohorts with HIV-infected pregnant women [25,77,80,81].

Madhi et al. performed a post hoc analysis of two randomized-controlled trials (RCTs) evaluating TIV efficacy among HIV-infected and HIV uninfected pregnant women for up to 24 weeks postpartum. More specifically, the authors assessed the correlation between the presence of HAI titers and protection provided against PCR-confirmed influenza illness. The share of HIV-uninfected and HIV-infected women with PCR-confirmed influenza illness was 5.6% and 20.5%, respectively [25,79]. The serologically diagnosed influenza virus infection (SDI) incidence was 35% and 43.6% in HIV-uninfected and HIV-infected women, respectively. These findings suggest that pregnant women living with HIV may not be as well-protected as HIV-uninfected pregnant women [25,79]. Future studies should consider evaluating baseline immunological responses and identifying the additional viral or bacterial co-infections to understand their impact in shaping serological responses to natural influenza infection and vaccination.

Previous studies revealed that TIV induces moderate to low HAI responses in people living with HIV, although with some conflicting results [77,79,80,81,82]. To that end, Nunes et al. decided to evaluate the immunogenicity and safety of different dosing schedules of TIV in pregnant women living with HIV [83]. The authors recruited pregnant women with HIV in a double-blind RCT. Using a computer-based randomization tool, authors were able to separate recipients of the TIV. The groups received the TIV containing 15 μg of each HA protein representative of three seasonal influenza strains for that year, through a single dose, double dose, or two single doses one month apart. The authors hypothesized that increasing the antigen in this cohort would increase the immunogenicity of the vaccine and therefore, increase the HAI titers in HIV-infected pregnant women. 

HAI titers were measured at enrollment and at one-month post-vaccination. The authors found that a higher proportion of women in the double-dose group had increased HAI titers to each strain compared to the single-dose group. In groups that were allocated a single dose or a double dose, HAI titers were measured within seven days of birth in the neonates. Infants born to mothers in the double-dose group had statistically significantly higher HAI geometric mean titers, and a higher proportion had titers of 1:40 or higher for A/H1N1pdm09 than those born to mothers in the single-dose group; a titer considered to be seroprotective. Overall, the study showed that a double dose of TIV moderately improved the immunogenicity in pregnant women with HIV, although no difference was observed in the incidence of symptomatic influenza in women or their infants [83]. 

To the knowledge of the authors, this was the first RCT that measured the immunogenicity of 3 different schedules for TIV dosing in pregnant women living with HIV. Interestingly, these results agree with the previous murine studies that are summarized above, whereby a prime-boost method with a TIV platform leads to an increase in the number of antibody titers in pregnant dams and their offspring that is more advantageous than using a single dose [57]. Thus, strengthening the use of a pregnant mouse model to evaluate universal influenza vaccine candidates. Future studies should consider evaluating TIV-induced vaccine efficacy in the context of additional high-risk factors such as pregnant women with metabolic syndrome [Reviewed in [84]]. 

While maternal influenza vaccination confers protection to mothers and their infants from laboratory-confirmed influenza, there is scarce information on whether the protection varies by gestational age [21,25,26,85]. Katz et al. investigated this question by recruiting women of childbearing age that were actively surveilled for pregnancy, consented, and subsequently randomized to receive maternal influenza vaccination or placebo with randomization stratified based on gestational age (17–25 or 26–34 weeks of gestation) [85]. Although they were not found to be statistically significant, cord blood and maternal antibody titers were generally present at greater levels in pregnancy [85]. However, the limitations in this study included the self-reporting of influenza-like-illness, which constitutes a potential source of bias. Additionally, it is essential to consider how pre-existing immunity, either from natural infection or vaccination, may also influence these results. 

### 4.2. Infant Protection as a Result of Maternal Influenza Vaccination 

While active immunization is the most efficient way to prevent influenza disease severity, current immunization strategies are not licensed for use in infants under six months old. Passive protection provided through transplacental transfer of maternal antibodies is the best strategy to achieve infant protection until they reach the appropriate age for vaccination [21,25,26,74]. To this end, previous studies have shown that the influenza vaccination of pregnant women protects their young infants against laboratory-confirmed influenza infection [21,25,26]. However, an infection with influenza virus might still predispose infants to subsequent bacterial infections that can result in severe pneumonia, a known cause of mortality during early life [86,87]. Thus, Nunes et al. interrogated the protective effect of maternal vaccination from all-cause lower-respiratory tract (ACLRT) infections in young infants [74]. The infants monitored in this study were born to women who participated in a double-blind placebo-controlled randomized control trial in 2011 and 2012, evaluating the efficacy of the TIV [25]. Infants in this study were monitored throughout the first six months of life [74]. 

The authors found that an influenza vaccination during pregnancy decreased ACLRT hospitalization during the first three months of life. These findings stood in stark contrast with findings from the placebo group, in which two-thirds of the ALRI hospitalizations occurred during the first three months of life. These results suggest that the benefit of maternal vaccination and protecting against influenza virus infection during early infancy might extend beyond simply protecting against an influenza-confirmed illness [74]. Other retrospective studies have not found such associations between influenza vaccination during pregnancy and the rates of acute respiratory illness among infants [88,89]. However, these studies might have been limited by the study design or the low rates of hospitalization in the infants [74,88,89]. Overall, given that a maternal influenza vaccination can prevent laboratory-confirmed influenza in infants younger than six months old, further longitudinal studies following mother-child dyads should consider investigating whether a maternal influenza vaccination can limit the likelihood of acquiring other known acute respiratory infections that remain a common cause of mortality in young infants such as respiratory syncytial virus (RSV) and bacterial pneumonia [21,25,26,87,88,89]. 

Evaluating the period during which infants can be protected through maternal vaccination has vital implications since there are currently no influenza vaccines licensed for use in infants younger than six months of age. Besides the ability to evaluate the inactivated vaccine efficacy in preventing ACLRT hospitalization in young infants, another study that used these data questioned the longevity of protection against influenza illness conferred by maternal vaccination. To do this, authors followed infants born to women who participated in the same RCT as described above during the first six months of life [21,25]. Overall, the vaccine efficacy was the highest among infants eight weeks (or two months) of age or younger, and the percentage of infants with seroprotective titers decreased from birth to 6 months.

The percentage of infants born to TIV recipient mothers with HAI titers of 1:40 or more at birth was 78.3, 56.6, and 81% for A/CA/09 H1N1 (H1N1pdm), A/H3N2, and B/Victoria strain, respectively. At 16 weeks old (or four months old), these numbers decreased substantially to 39.5%, 19.1%, and 40% for A/CA/09 H1N1 (H1N1pdm), A/H3N2, and B/Victoria, respectively. These numbers were further reduced at 24 weeks old (or six months old) to 10.0%, 6.7%, and 9.4% for A/CA/09 H1N1 (H1N1pdm), A/H3N2, and B/Victoria, respectively. This highlights that the concentration of maternally acquired antibodies conferred by maternal immunization in young infants decreases after birth. By 16 weeks (or four months of age), less than 40% of the infants born to TIV-vaccinated mothers had HAI titers of 1:40 or more. However, the authors only evaluated the functionality of the antibodies by performing HAIs. Future studies should assess the direct comparisons between microneutralization assays and an antibody-mediated cytotoxicity assay (ADCC). The authors also concluded that most antibodies detected via HAIs were acquired via transplacental transport. However, the authors did not test or evaluated secretory IgA, which is predominantly transferred through breast milk. Hence, it is difficult to conclude that only vertically transferred antibodies mediate the observed protection. Future studies should consider evaluating the prevalence of both IgG and IgA to isolate the proportion of functional antibodies transferred to the infant. Alongside understanding antibody-mediated protection in these cohorts, cellular-mediated immunity, including the transfer of maternal immune cells, should be considered when testing different vaccination platforms in these populations. 

## 5. Conclusions

Pregnancy has been identified as a high-risk factor for severe influenza disease for more than a century. However, studies are still underway to better understand the mechanisms that mediate the increased influenza pathogenicity observed during pregnancy [Reviewed in [53]]. Given these data, protecting this population through vaccination becomes crucial. This is also true of newborns and infants. Given the vaccination gap during an infant’s first months of life, it is desirable to confer a robust passive immune response to them via a transplacental transfer of maternal antibodies while in utero or through breast milk post-birth. Although ideal, performing large-scale vaccination studies in humans with longitudinal sampling can be challenging. To bridge this gap in knowledge, animal models of pregnancy have provided an opportunity to establish the scientific premise and move influenza vaccine candidates to the clinic.

Murine studies showed that while the standard of care platforms such as TIV can provide some baseline protection against a homologous lethal influenza challenge, other platforms, including MN patches may elicit a longer-lasting antibody response in the offspring, which is capable of protecting against influenza challenge until six weeks of age. While interesting, additional studies should include data showing the safety of innovative influenza vaccine platforms, specifically with regards to immunogenicity in pregnant dams. Other studies using pregnant ferrets showed that naïve kits had higher levels of H1N1pdm specific antibodies compared to kits that had a passive transfer of maternal antibodies via natural infection. Interestingly, this result was only true upon homologous challenge. When infected with a different strain or sub-type, serological responses were higher, even in kits that had the passive transfer of H1N1pdm specific maternal antibodies [70]. 

Since studies using mice and ferrets observed that protection is limited to strains homologous to that of the vaccine, it would be interesting to evaluate other vaccine platforms that could elicit a broader antibody response capable of protecting against heterologous influenza challenge. While we focused on serological responses, including antibodies to the antigen of interest, future studies should also evaluate the presence of maternal immune cells in the offspring, otherwise known as maternal microchimerisms [90,91]. To this end, while studies using mice will provide valuable information to take influenza vaccine candidates further, it will be important to test new influenza vaccine candidates in other biologically relevant models such as the ferret or non-human primates. 

Studies that evaluate the safety, immunogenicity, and efficacy of the TIV platform in pregnant women cohorts have found that it is protective against PCR-confirmed influenza illness, however, it is not clear how severe the symptoms were in these cases. Studies have also concluded that infants born to HIV-infected pregnant women have a lower proportion of HAI titers as compared to infants born to HIV-uninfected pregnant women. These results highlight the need for the further consideration of baseline immunological responses and pre-existing viral infections when evaluating serological responses to influenza infection and vaccination in future cohorts. While TIV is a currently licensed vaccine and the standard of care for pregnant women, new data and initiatives seeking to ameliorate serological responses to influenza in this population have inspired optimism. Future studies should evaluate the breadth and depth of the antibody responses elicited by the TIV platform as compared to the new influenza vaccine platforms and candidates, as well as passive immunity to and protection from infection in the offspring.
